# The Effect of Maternal Haemoglobinopathies and Iron Deficiency Anaemia on Foetal Growth Restriction: A Systematic Review and Meta‐Analysis

**DOI:** 10.1111/mcn.13787

**Published:** 2025-04-15

**Authors:** Rajeev Jayalakshmi, Shilpa Gaidhane, Suhas Ballal, Sanjay Kumar, Mahakshit Bhat, Shilpa Sharma, M. Ravi Kumar, Sarvesh Rustagi, Mahalaqua Nazli Khatib, Nishant Rai, Sanjit Sah, Sorabh Lakhanpal, Hashem Abu Serhan, Ganesh Bushi, Muhammed Shabil

**Affiliations:** ^1^ Department of Public Health and Community Medicine Central University of Kerala Tejaswini Hills, Periye Kasaragod Kerala India; ^2^ One Health Centre, Datta Meghe Institute of Higher Education, Jawaharlal Nehru Medical College Wardha India; ^3^ Department of Chemistry and Biochemistry School of Sciences JAIN (Deemed to be University) Bangalore Karnataka India; ^4^ Department of Allied Healthcare and Sciences Vivekananda Global University Jaipur Rajasthan India; ^5^ Department of Medicine National Institute of Medical Sciences NIMS University Rajasthan Jaipur India; ^6^ Chandigarh Pharmacy College, Chandigarh Group of Colleges‐Jhanjeri Mohali Punjab India; ^7^ Department of Chemistry Raghu Engineering College Visakhapatnam Andhra Pradesh India; ^8^ School of Applied and Life Sciences Uttaranchal University Dehradun Uttarakhand India; ^9^ Division of Evidence Synthesis Global Consortium of Public Health and Research, Datta Meghe Institute of Higher Education Wardha India; ^10^ Department of Biotechnology Graphic Era (Deemed to be University) Dehradun India; ^11^ Department of Allied Sciences Graphic Era Hill University Dehradun India; ^12^ Department of Paediatrics Dr. D. Y. Patil Medical College, Hospital and Research Centre, Dr. D. Y. Patil Vidyapeeth (Deemed‐to‐be University) Pune Maharashtra India; ^13^ Department of Medicine Korea Universtiy Seoul South Korea; ^14^ School of Pharmaceutical Sciences Lovely Professional University Phagwara India; ^15^ Department of Ophthalmology Hamad Medical Corporation Doha Qatar; ^16^ Center for Global Health Research, Saveetha Institute of Medical and Technical Sciences, Saveetha Medical College and Hospital Saveetha University Chennai India; ^17^ Evidence for Policy and Learning, Global Center for Evidence Synthesis Chandigarh India; ^18^ University Center for Research and Development Chandigarh University Mohali Punjab India; ^19^ Medical Laboratories Techniques Department AL‐Mustaqbal University Hillah Babil Iraq

**Keywords:** anaemia, good health and well‐being, intrauterine growth restriction, meta‐analysis

## Abstract

Maternal anaemia is a significant global health issue that adversely affects both maternal and foetal outcomes, particularly, intrauterine growth restriction (IUGR). This systematic review and meta‐analysis aimed to consolidate existing evidence on the impact of maternal anaemia on the risk of IUGR. We conducted a comprehensive search across PubMed, Embase, Cochrane and Web of Science until 28 February 2024. Eligible studies included observational designs that reported maternal anaemia and its association with IUGR or small for gestational age (SGA) outcomes. The pooled odds ratios (ORs) were calculated using a random‐effects model and heterogeneity was assessed with the *I*² statistic. The R software (version 4.3) was used for statistical analyses. A total of 38 studies involving 3,871,849 anaemic and 27,978,450 non‐anaemic pregnant women were included. The pooled analysis demonstrated that anaemia in pregnancy is associated with a significantly increased risk of IUGR (OR = 1.30, 95% CI: 1.05–1.62, *I*² = 97%). Subgroup analyses by anaemia severity showed non‐significant associations for mild (OR = 0.84, 95% CI: 0.58–1.23) and moderate anaemia (OR = 0.98, 95% CI: 0.48–1.98), while severe anaemia indicated a higher, though non‐significant, risk of IUGR (OR = 1.42, 95% CI: 0.69–2.93). Maternal anaemia is associated with a heightened risk of IUGR, highlighting the critical need for effective management and early intervention strategies within prenatal care settings. Future research should focus on elucidating the effects of different severities of anaemia on birth outcomes, including IUGR and long‐term effects later in life.

## Summary

- Increased IUGR risk: Maternal anaemia is linked to a 30% increase in the odds of IUGR.
- Severity impact: No significant IUGR risk with mild or moderate anaemia; severe anaemia shows a higher, yet non‐significant, risk.
- Result variability: Substantial heterogeneity (*I*² = 97%) suggests wide variability in the anaemia–IUGR association across different studies.
- Haemoglobinopathy effects: No significant link between haemoglobinopathies (sickle cell, thalassaemia) and IUGR, though results are highly variable.
- Management needs: Emphasizes early anaemia detection and management in pregnancy, suggesting targeted nutritional support and possibly intravenous iron.

## Graphical abstract

This meta‐analysis demonstrates a significant association between maternal anaemia and a 30% increased risk of intrauterine growth restriction (IUGR). These findings emphasize the need for early identification and effective management of anaemia during pregnancy to improve foetal outcomes.

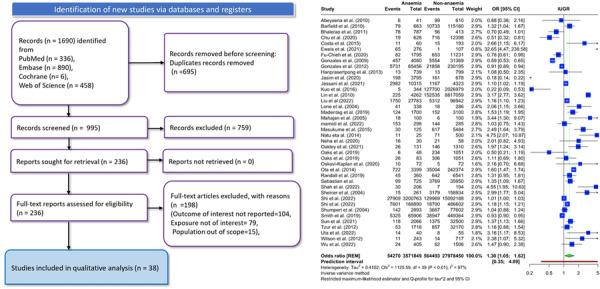

## 1 Introduction

Anaemia is characterized by a deficiency in red blood cells or haemoglobin, consequently affecting the oxygen‐carrying capacity and the ability to meet the body's physiologic needs (Weyand et al. 2023; Karami et al. 2022). The global prevalence of anaemia across all ages in 2021 was 24.3%, constituting 1.92 billion people (Gardner et al. 2023). Maternal anaemia is a major health concern affecting expectant mothers worldwide (Weyand et al. 2023; Karami et al. 2022). Anaemia during pregnancy is often caused by nutritional deficiencies, chronic diseases or other factors and can have far‐reaching implications for maternal health and foetal development (Finkelstein et al. 2020; Dorsamy, Bagwandeen, and Moodley 2022). Maternal anaemia can result from various factors, the most common being iron deficiency anaemia. This occurs when the body lacks an adequate supply of iron to produce sufficient haemoglobin, the protein responsible for transporting oxygen in the blood (Garzon et al. 2020). Other causes include vitamin deficiencies (e.g., folate or vitamin B12), chronic medical conditions (such as thalassaemia or sickle cell disease) and dietary habits lacking essential nutrients (Stephen et al. 2018). Anaemia in pregnancy can lead to several adverse effects on the mother's health. Fatigue, weakness and decreased energy levels are common symptoms, making it challenging for pregnant women to cope with the physical demands of pregnancy (Helmy, Elkhouly, and Ghalab 2018).

Haemoglobin concentration thresholds for classifying anaemia by severity for pregnant women aged ≥ 15 years are as follows—mild (100–109 g/L), moderate (70–99 g/L) and severe (< 70 g/L) (Shi et al. 2022). In 2019, more than one‐third of pregnant women were anaemic worldwide (Stevens et al. 2022; World Health Organization 2021). Anaemic mothers are at a higher risk of developing complications such as infections, pre‐eclampsia and postpartum haemorrhage, which can further endanger their well‐being (Barut and Mohamud 2023). The consequences of maternal anaemia extend beyond the mother's health and directly affect foetal development. Insufficient oxygen delivery to the developing foetus can result in intrauterine growth restriction (IUGR), low birth weight and premature birth (Sharma, Shastri, and Sharma 2016). These factors increase the risk of neonatal complications, including respiratory distress syndrome, infections and long‐term developmental issues (von Beckerath et al. 2013; Gilbert and Danielsen 2003; Takahashi et al. 2021; Reddy et al. 2021).

Foetal growth restriction (FGR), also known as IUGR, occurs when a foetus does not reach its expected growth potential in the womb (Nardozza et al. 2017). This condition is typically diagnosed when an estimated foetal weight falls below the 10th percentile for gestational age. Clinically, infants affected by FGR are often classified as small for gestational age (SGA), meaning their birth weight is below the 10th percentile for their gestational age at birth. FGR can be categorized further: moderate FGR is when the birth weight is between the 3rd and 10th percentiles and severe FGR is when it is below the 3rd percentile (Dall'Asta et al. 2017; Pels et al. 2020). Studies use IUGR and SGA interchangeably, though there are minute differences between the two. FGR is a relatively common complication of pregnancy, and in most cases, the foetuses are born small but healthy (Pels et al. 2020). True FGR is due to placental insufficiency in delivering adequate nutrients and oxygen to the developing foetus, resulting in stunted growth. As the FGR in utero most often goes undetected, the neonatal description of SGA is the most used proxy for the FGR (Damhuis, Ganzevoort, and Gordijn 2021).

Suboptimal growth in foetuses diagnosed with IUGR is linked to heightened risks of mortality and morbidity around the time of birth. Significant outcomes of IUGR encompass stillbirth and adverse neuro‐developmental trajectories during childhood (Benítez Marín et al. 2022; Unterscheider et al. 2014), along with a heightened risk of developing chronic conditions such as diabetes, hypertension and cardiovascular diseases in adulthood (Huang et al. 2018). Issues related to emotional, behavioural and social challenges in later life have also been observed (Jee et al. 2023; Shah et al. 2024). Furthermore, IUGR increases the likelihood of various neonatal complications, including meconium aspiration syndrome (MAS), hypoglycaemia, hyaline membrane disease (HMD), early onset sepsis (EOS) and intrapartum asphyxia (Dini et al. 2024; Rodrigues et al. 2022; Minuye Birihane et al. 2021; Köstlin‐Gille et al. 2021).

There are previous systematic reviews and meta‐analyses on the effect of maternal anaemia on adverse pregnancy outcomes in general (Rahman et al. 2016). However, systematic reviews and meta‐analyses addressing IUGR specifically are limited. In this study, we systematically reviewed and analyzed the literature on maternal anaemia and IUGR/FGR.

## 2 Methods

We conducted a systematic review and meta‐analysis of observational studies. This study was conducted in conformity with PRISMA 2020 (Supporting Information S1: Table S1) (Page et al. 2021). In addition, the study protocol was prospectively registered in the International Prospective Register of Systematic Reviews (https://www.crd.york.ac.uk/prospero/; registration number CRD42024507906).

### 2.1 Eligibility Criteria

For this systematic review and meta‐analysis, we considered all observational studies, regardless of their design (including cross‐sectional, case–control and cohort studies) that reported on the specified exposure (maternal anaemia) and the outcome of interest (IUGR/SGA). There were no restrictions based on geographic location (whether rural or urban) or the setting of the study (such as community‐based, healthcare facility or workplace environments). Only studies published as full‐text articles were included in the analysis. We excluded abstracts presented at conferences, case reports, case series and unpublished manuscripts.

### 2.2 Literature Search

An electronic literature search was performed in various databases, including PubMed, Embase, Cochrane and Web of Science, up to 27 November 2023 and later updated on 28 February 2024. MeSH terms and keywords related to anaemia and IUGR or SGA were used in the search strategy. No restrictions were imposed on the search based on the date of publication or article type. The complete search strategy is displayed in Supporting Information S1: Table S2.

### 2.3 Study Selection

The process of selecting studies for inclusion in this systematic review and meta‐analysis was conducted in three phases. Initially, two independent researchers screened titles, abstracts and keywords after performing the initial literature search to identify potentially relevant studies. Studies that preliminarily met the inclusion criteria had their full texts retrieved for more thorough review. In the second phase, these full texts were carefully examined by the same researchers to ensure they met all specified inclusion criteria regarding study design, participant characteristics, exposures and outcomes of interest. In the final phase of selection, any discrepancies between the investigators regarding study inclusion were discussed and resolved with the assistance of a senior researcher. If any essential details were absent in the studies, the corresponding authors were contacted for clarification or additional data. Studies were excluded from the analysis if the authors did not respond or the required information remained unavailable. This structured approach ensured a rigorous and unbiased selection of studies for the analysis.

### 2.4 Data Extraction

Data for this systematic review and meta‐analysis were meticulously gathered using a structured data extraction form designed to capture essential details of each study. This form collected general information such as the author's name, year of publication, study population characteristics, type of anaemia studied, event counts for the outcomes in both anaemic and non‐anaemic groups, definitions of anaemia employed, effect sizes reported and methodological details including study design, setting, sample size and methods for assessing exposure and outcomes.

To evaluate the quality of the studies included in this analysis, two independent reviewers applied the Newcastle–Ottawa Quality Assessment Scale, which is detailed in Supporting Information S1: Table S3. This scale evaluates three critical domains: selection (with a maximum of four stars), comparability (up to two stars) and outcome (up to three stars). Criteria within these domains include the representativeness of the study sample, justification of the sample size, response rates, accuracy of exposure assessment, control measures for confounding variables, outcome assessment techniques and the statistical tests used. The total possible score for each study can range from zero to nine stars, indicating the study's overall quality from low to high.

### 2.5 Statistical Analysis

We calculated the pooled odds ratio (OR) for IUGR with the presence of anaemia by combining the total number of anaemic and non‐anaemic participants together with the IUGR outcome in each group. A random‐effects model was employed for the meta‐analysis. Given the expected heterogeneity among the studies, a random‐effects meta‐analytical model was used. This model accounts for variation both within and between studies, making it suitable for clinical and epidemiological data. The *I*² statistic was used to evaluate heterogeneity among the studies, with values greater than 50% indicating substantial heterogeneity (Gandhi et al. 2023; Pandey, Shabil, and Bushi 2024). Subgroup analyses were conducted based on the severity of anaemia. Publication bias was assessed using funnel plot and Egger's test. Sensitivity analyses were conducted to evaluate the robustness and reliability of the findings. Meta‐regression was conducted to assess the effect of variables on the overall result. All statistical analyses were performed using R software (version 4.3), employing the ‘meta’ and ‘metafor’ packages for performing meta‐analysis (Shamim et al. 2023).

## 3 Results

### 3.1 Literature Search

**Figure 1:**
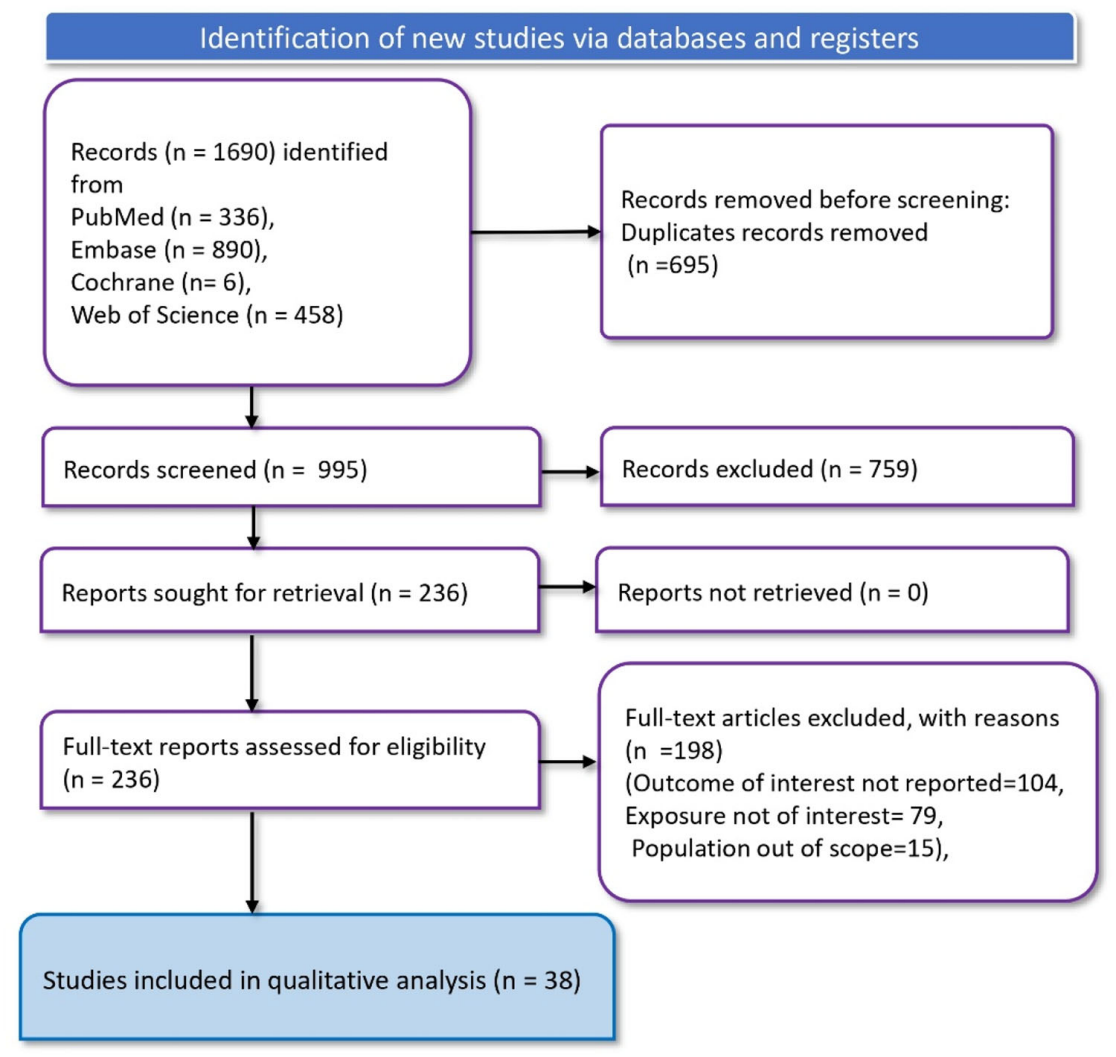
PRISMA flow diagram depicting article screening and screening process.

We identified a total of 1690 records through database searches. Upon removal of 695 duplicate records, 995 records were screened, resulting in the exclusion of 759 records. Subsequent retrieval of reports yielded 236 records for which full‐text articles were assessed for eligibility. Of these, 198 full‐text articles were excluded for the following reasons: the outcome of interest was not reported in 104 articles, the exposure of interest was not reported in 79 articles and the population was out of scope in 15 articles. Ultimately, 38 studies met the eligibility criteria and were included in the meta‐analysis (Shi et al. 2022; Col Madendag et al. 2019; Lone, Qureshi, and Emmanuel 2004; Jessani et al. 2021; Smith et al. 2019; Abeysena, Jayawardana, and Seneviratne 2010; Joshi et al. 2011; Sebastian et al. 2015; Chu et al. 2020; Hanprasertpong and Hanprasertpong 2015; Jasim, Al‐Momen, and Al‐Asadi 2020; Liu et al. 2022; Kuo and Caughey 2016; Natu et al. 2014; Costa, Viana, and Aguiar 2015; Gonzales et al. 2012; Lin et al. 2010; Barfield et al. 2010; Chu et al. 2020; Eweis et al. 2021; Mahajan et al. 2005; Masukume et al. 2015; Neha et al. 2020; Oakley et al. 2022; Oaks et al. 2019; Oskovi‐Kaplan et al. 2021; Ota et al. 2014; Sheiner et al. 2004; Shumpert, Salihu, and Kirby 2004; Sun et al. 2021; Tzur et al. 2012; Uta et al. 2022; Wilson et al. 2012; Wu et al. 2022; Mamidi et al. 2022; Shah et al. 2022; Randall et al. 2019; Gonzales, Steenland, and Tapia 2009) (Figure 1).

### 3.2 Characteristics of Included Studies

The important characteristics of the included studies are displayed in Table 1. The included studies were observational, predominantly consisting of cross‐sectional, retrospective and prospective cohort designs. Nested case–control study and secondary analysis of trials were also present. The studies originated from various countries, with four from China, six from India, two from Turkey, two from Pakistan, one from Canada, three from Taiwan, one from Thailand, one from Iraq, two from Peru, three from the United States, one from Brazil, one from Egypt, one from Australia, one from Ghana, one from Romania, one from Sri Lanka, three involving multiple countries, two from Israel, one from Malawi and one from the United Kingdom. Participant mean ages ranged from 21 to 37 years, predominantly reflecting an adult female demographic within obstetrical and gynaecological contexts. The primary focus was iron deficiency anaemia, although several studies also assessed sickle cell anaemia and thalassaemia. Some studies provided risk estimates quantifying the associations between various factors and anaemia. The diagnostic criteria for anaemia classification varied, with most studies using haemoglobin levels as a threshold and establishing specific ranges for mild, moderate and severe anaemia. Nevertheless, the thresholds for these levels showed slight variations among the studies.

**Table 1:** Characteristics of included studies.

Study	Study design	Country	Sampling cluster	Sample size	Mean age (years)	Type of anaemia	OR/RR/PR (95% CI) for anaemia	Anaemia definition
Abeysena, Jayawardana, and Seneviratne (2010)	Prospective cohort study	Sri Lanka	Two medical offices of health areas	817	27.03	Iron deficiency anaemia	1.24 (0.88−1.74)	Hb < 11 g/dL
Barfield et al. (2010)	Retrospective cohort study	United States	Massachusetts pregnancy to early life longitudinal (PELL) data system	84,561	NA	Sickle cell	1.3 (1.1–1.5)	Sickle cell anaemia
Joshi et al. (2011)	Prospective cohort study	India	Obstetric outpatient department of a rural tertiary care hospital, Nagpur, India	1200	24.63	Iron deficiency anaemia	1.3	Hb < 11 g/dL
Chu et al. (2020)	Retrospective cohort study	Taiwan	Taipei Chang Gung Memorial Hospital's computerized obstetrics database	15,602	NA	Iron deficiency anaemia	0.56 (0.35–0.89)	Hb level below the 10th percentile
Costa, Viana, and Aguiar (2015)	Prospective observational study	Brazil	Odilon Behrens Municipal Hospital and Hospital of UFMG	245	NA	Sickle cell	2.66 (1.15–6.17)	NA
Eweis et al. (2021)	Cross‐sectional study	Egypt	Beni‐Suef University Hospital	383	28.75	Iron deficiency anaemia	25.2 (3.4–184.1)	Mild: Hb 10–10.9 g/dL, moderate: Hb 7–9.9 g/dL, severe: Hb < 7.0 g/dL
Chu et al. (2020)	Retrospective cohort study	Taipei	Taipei Chang Gung Memorial Hospital from 2001 to 2016	15,602	NA	Iron deficiency anaemia	0.84 (0.66–1.06)	Hb < 10.8 g/dL
Gonzales, Steenland, and Tapia (2009)	Cohort study	Peru	Perinatal Information System (PIS) database	35,449	NA	Iron deficiency anaemia	NA	NA
Gonzales et al. (2012)	Secondary analysis	Peru	SIP in Spanish over the period 2000–2010	295,651	NA	Iron deficiency anaemia	NA	Ranges of Hb levels from < 7 to 10–10.9 g/dL
Hanprasertpong and Hanprasertpong (2015)	Retrospective case‐controlled and cohort study	Thailand	Maternal and Fetal Medicine Unit, Songklanagarind Hospital database	240	NA	Thalassaemia	1.05 (0.49–2.26)	Hb < 11 g/dL
Jasim, Al‐Momen, and Al‐Asadi (2020)	Cross‐sectional study	Iraq	Department of Obstetrics and Gynecology at Medical City Hospital in Baghdad	4473	24.54	Iron deficiency anaemia	NA	Mild anaemia: Hb = 10–10.9 g/L, moderate anaemia: Hb = 7.1–9.9 g/L, severe anaemia: Hb < 7 g/L
Jessani et al. (2021)	Secondary analysis of trial	Multiple sites	ASPIRIN Trial	11,976	21.00	Iron deficiency anaemia	NA	Hb levels: 70–89 (severe), 90–109 (moderate), 110–129 (mild), ≥ 130 g/L (normal)
Kuo and Caughey (2016)	Retrospective cohort study	United States	California Vital Statistics Birth Certificate Data, infant Vital Statistics Death Certificate Data, California Patient Discharge Data, and Vital Statistics Fetal Death Files	2,027,323	NA	Sickle cell	1.96 (1.18−3.25)	NA
Lin et al. (2010)	Observational study	Taiwan	National Health Research Institute in Taiwan	196	NA	Iron deficiency anaemia	1.44 (0.91–2.27)	NA
Liu et al. (2022)	Prospective observational study	China	Chinese Ministry of Health database	124,725	24.37	Iron deficiency anaemia	1.13 (1.07–1.20)	Mild anaemia: 100 ≤ Hb < 110 g/L, moderate‐to‐severe anaemia: Hb < 100 g/L
Lone, Qureshi, and Emmanuel (2004)	Cohort study	Pakistan	Obstetrics Department of the Aga Khan University Hospital Karachi, Pakistan	629	26.96	Iron deficiency anaemia	1.9 (1.1–3.3)	Hb < 11 g/dL
Col Madendag et al. (2019)	Retrospective cohort study	Turkey	Ayseri City Hospital between June 2018 and July 2019	4800	26.05	Iron deficiency anaemia	NA	Severe anaemia: Hb < 7 mg/dL, moderate anaemia: Hb: 7–9.9 mg/dL, mild anaemia: Hb: 10–10.9 mg/dL. Iron deficiency anaemia: serum ferritin level < 15 mcg/L without infection
Mahajan et al. (2005)	Observational study	India	AIIMS, New Delhi	300	23.70	Iron deficiency anaemia	NA	Hb < 11 g/dL
Mamidi et al. (2022)	Prospective cohort study	India	Slum in Hyderabad, India	1061	23.00	Iron deficiency anaemia	NA	Hb level: 10.73– < 11 g/dL
Masukume et al. (2015)	Prospective cohort study	New Zealand, Australia, England and Ireland	SCOPE (Screening for Pregnancy Endpoints)	5609	28.98	Iron deficiency anaemia	0.85 (0.5–1.46)	Hb < 11 g/dL
Natu et al. (2014)	Retrospective study	India	Obstetric ward of a tertiary care centre at Indore, Madhya Pradesh, India	2068	24.20	Sickle cell	9.2 (3.5–24.6)	NA
Neha et al. (2020)	Nested case–control study	India	Kasturba medical college, Manipal	88	25.75	Iron deficiency anaemia	1.47 (0.91–2.38)	Hb < 11 g/dL
Oakley et al. (2022)	Cohort study	United Kingdom	Guy's and St Thomas' NHS Foundation Trust	1441	NA	Sickle cell	2.62 (1.82–3.78)	Sickle cell anaemia
Oaks et al. (2019)	Cohort study	Malawi	International Lipid‐Based Nutrient Supplements Project	2380	25.30	Iron deficiency anaemia	1.38 (0.99–1.91)	Hb < 70 g/L at baseline or < 100 g/L at 36 weeks of gestation
Oskovi‐Kaplan et al. (2021)	Retrospective cohort study	Turkey	Zekai Tahir Burak Women's Health Training and Research Hospital	72	26.65	Iron deficiency anaemia	0.73 (0.58–0.93)	Hb < 10 g/dL with treatment and Hb < 10 g/dL without treatment
Ota et al. (2014)	Cross‐sectional study	Multicounty	HO Multi‐Country Survey on Maternal and Newborn Health	295,829	NA	Iron deficiency anaemia	NA	Hb < 7 g/L
Randall et al. (2019)	Cross‐sectional study	Australia	Two large tertiary public hospitals in NSW, Australia	7104	NA	Gestational anaemia	NA	Groups based on changing Hb levels from 1 to 3 trimesters
Sebastian et al. (2015)	Cross‐sectional study	India	Labour room register: 1996–2010	202,525	30.00	Iron deficiency anaemia	1.29 (1.01–1.65)	Hb < 11 g/dL
Shah et al. (2022)	Cross‐sectional study	Pakistan	Liaquat University of medical and health sciences Jamshoro	400	37.02	Iron deficiency anaemia	NA	Hb < 11 g/dL
Sheiner et al. (2004)	Retrospective cohort study	Israel	Soroka University Medical Center	159,195	NA	Thalassaemia	2.4 (1.4–4.2)	Hb < 11 g/dL
Shi et al. (2022)	Retrospective cohort study	China	China's Hospital Quality Monitoring System	18,948,443	32.86	Iron deficiency anaemia	NA	Mild anaemia: Hb < 100–109 g/L, moderate anaemia: Hb 70–99 g/L, severe anaemia: Hb < 70 g/L
Shumpert, Salihu, and Kirby (2004)	Retrospective cohort study	United States	National Center for Health Statistics (NCHS)	80,495	NA	Iron deficiency anaemia	NA	Haemoglobin level < 10.0 g/dL or haematocrit < 30% during pregnancy
Smith et al. (2019)	Retrospective cohort study	Canada	British Columbia Perinatal Data Registry	515,270	NA	Iron deficiency anaemia	NA	Mild anaemia: Hb 9–10.9 g/dL, moderate anaemia: Hb 7–8.9 g/dL, severe anaemia: Hb < 7 g/dL
Sun et al. (2021)	Retrospective cohort study	China	International Peace Maternal and Child Health Hospital, Shanghai Jiao Tong University	46,578	31.02	Iron deficiency anaemia	1.46 (1.20–1.78)	Hb < 110 g/L in the first and third trimesters, and < 105 g/L in the second trimester
Tzur et al. (2012)	Retrospective cohort study	Israel	Soroka University Medical Center	33,888	29.07	Iron deficiency anaemia	1.98 (0.9–1.6)	Hb < 11 g/dL
Uta et al. (2022)	Retrospective cohort study	Romania	Timis County Emergency Clinical Hospital	446	NA	Iron deficiency anaemia	NA	Hb < 11.0 g/dL in the first trimester, < 10.5 g/dL in the second and third trimesters, and < 10.0 g/dL postpartum
Wilson et al. (2012)	Retrospective cohort study	Ghana	KBTH, Accra, Ghana	960	28.76	Sickle cell	NA	Hb < 11 g/dL
Wu et al. (2022)	Retrospective cohort study	China	Union Shenzhen Hospital of the Huazhong University of Science and Technology	1911	31.00	Iron deficiency anaemia	1.37 (0.78–2.39)	Grouper based on Hb levels < 110, 110–119, 120–130 g/L

Abbreviations: CI, confidence interval; Hb, haemoglobin; NA, not available; OR, odds ratio; PR prevalence ratio; RR, relative risk.

### 3.3 Association Between Maternal Anaemia and IUGR

**Figure 2:**
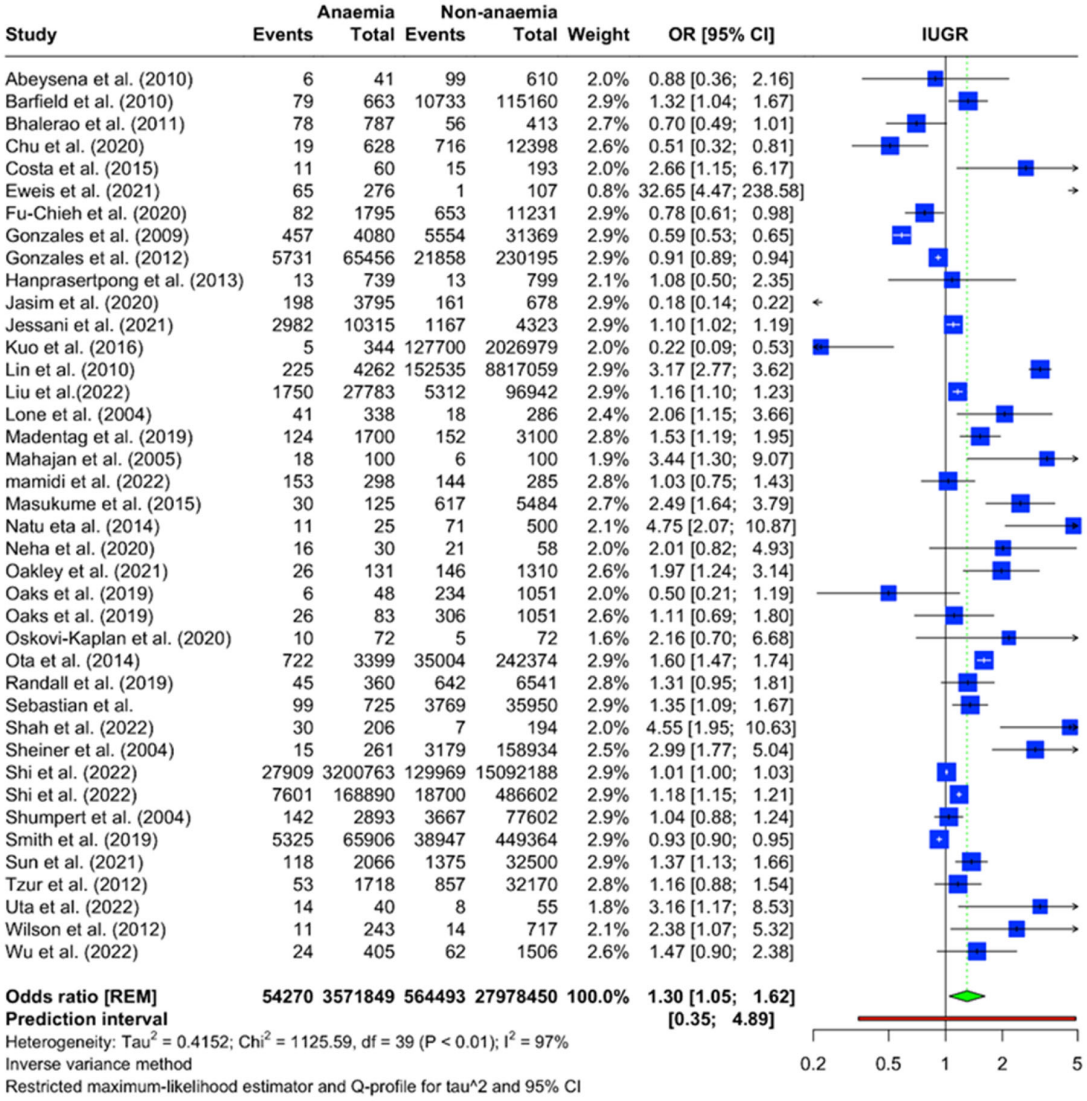
Forest plot illustrating the association of maternal anaemia and IUGR based on the meta‐analysis.

The forest plot presented in Figure 2 illustrates the findings of a meta‐analysis evaluating the relationship between anaemia and IUGR. The analysis included data from 38 studies involving 3,871,849 anaemic and 27,978,450 non‐anaemic pregnant women. The overall pooled OR for IUGR was determined to be 1.30 (95% CI: 1.05–1.62), indicating that anaemia is significantly associated with 30% increased odds of IUGR when compared to non‐anaemia. There was substantial heterogeneity observed among the studies (*I* ^2^ = 97%). The prediction interval ranged from 0.35 to 4.89, underscoring the wide variability of the effect sizes that future studies might report. This suggests that while the mean effect size signals an increased risk, the extent of this risk is likely to differ significantly across various populations and settings.

### 3.4 Levels of Anaemia Severity and IUGR

**Figure 3:**
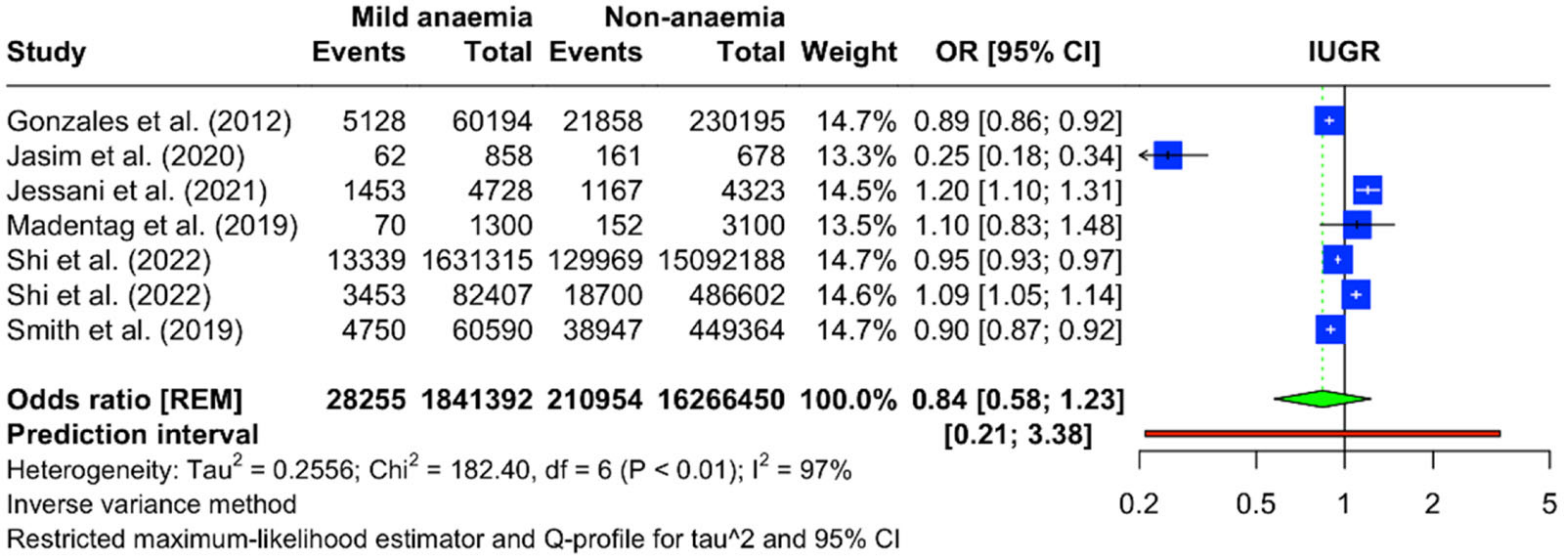
Forest plot illustrating the pooled OR for IUGR with mild maternal anaemia compared to non‐anaemia, based on the meta‐analysis.

**Figure 4:**
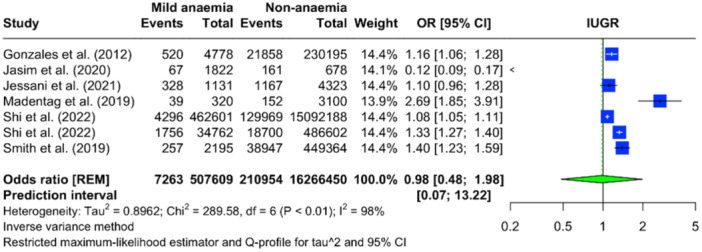
Forest plot illustrating the pooled OR for IUGR with moderate maternal anaemia compared to non‐anaemia, based on the meta‐analysis.

**Figure 5:**
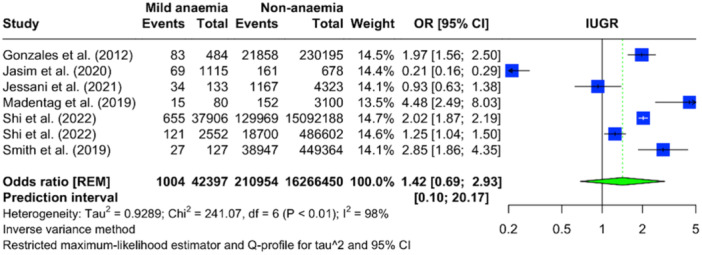
Forest plot illustrating the pooled OR for IUGR with severe maternal anaemia compared to non‐anaemia, based on the meta‐analysis.

Data from seven studies (only seven studies rereported severity levels for anaemia) were analyzed to evaluate the relationship between various severities of maternal anaemia and IUGR. In cases of mild maternal anaemia, there were 28,255 events among 1,841,392 individuals, with a pooled OR for IUGR of 0.84 (95% CI: 0.58–1.23), indicating no significant association (Figure 3). For moderate maternal anaemia, the same seven studies reported 7263 events among 507,609 individuals, yielding a pooled OR of 0.98 (95% CI: 0.48–1.98), which suggests no significant difference in the risk of IUGR compared with non‐anaemic individuals (Figure 4). The analysis for severe maternal anaemia included 1004 severe anaemic mothers from 423,977 individuals. In contrast, the non‐anaemia comparison group consisted of 210,954 events among 16,266,450 individuals. The pooled OR for severe anaemia was 1.42 (95% CI: 0.69–2.93), implying 42% increased odds of IUGR. However, due to the wide CI that includes the null effect value, this increase is not statistically significant (Figure 5). Significant heterogeneity was observed across all severity levels of anaemia, with an *I* ^2^ statistic of 97%–98%.

### 3.5 Association Between Maternal Haemoglobinopathy‐Related Anaemia and IUGR

**Figure 6:**
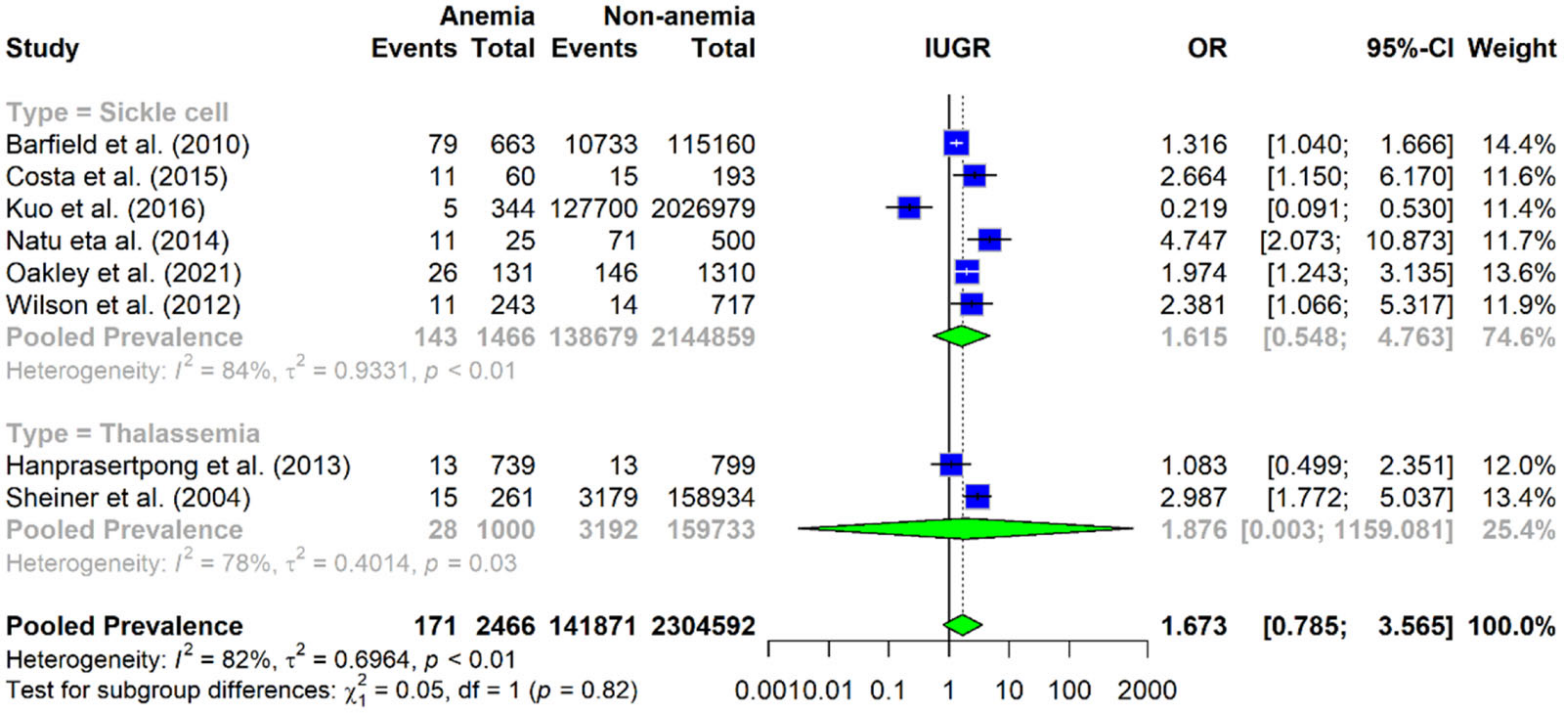
Forest plot illustrating the pooled OR for IUGR with haemoglobinopathy‐related anaemia, based on the meta‐analysis.

The analysis includes six studies on sickle cell anaemia and two studies on thalassaemia. The overall pooled OR for IUGR in mothers with sickle cell anaemia was 1.61 (95% CI: 0.548–4.763), indicating a non‐significant association between sickle cell anaemia and IUGR. There was considerable heterogeneity among the studies (*I*² = 84%, *p* < 0.001), suggesting that the effect of sickle cell anaemia on IUGR risk varies across different populations and settings. For thalassaemia, the pooled OR was 1.87 (95% CI: 0.003–1159.08) based on data from two studies, indicating that the result is not statistically significant. There was high heterogeneity among the studies (*I*² = 78%, *p* < 0.05). Overall, the pooled prevalence of IUGR for haemoglobinopathy‐related anaemia (sickle cell and thalassaemia combined) was 1.67 (95% CI: 0.79–3.55), demonstrating a non‐significant relation of IUGR in pregnancies affected by haemoglobinopathy‐related anaemia. Significant heterogeneity (*I*² = 82%) was observed across studies (Figure 6).

### 3.6 Sensitivity Analysis

**Figure 7:**
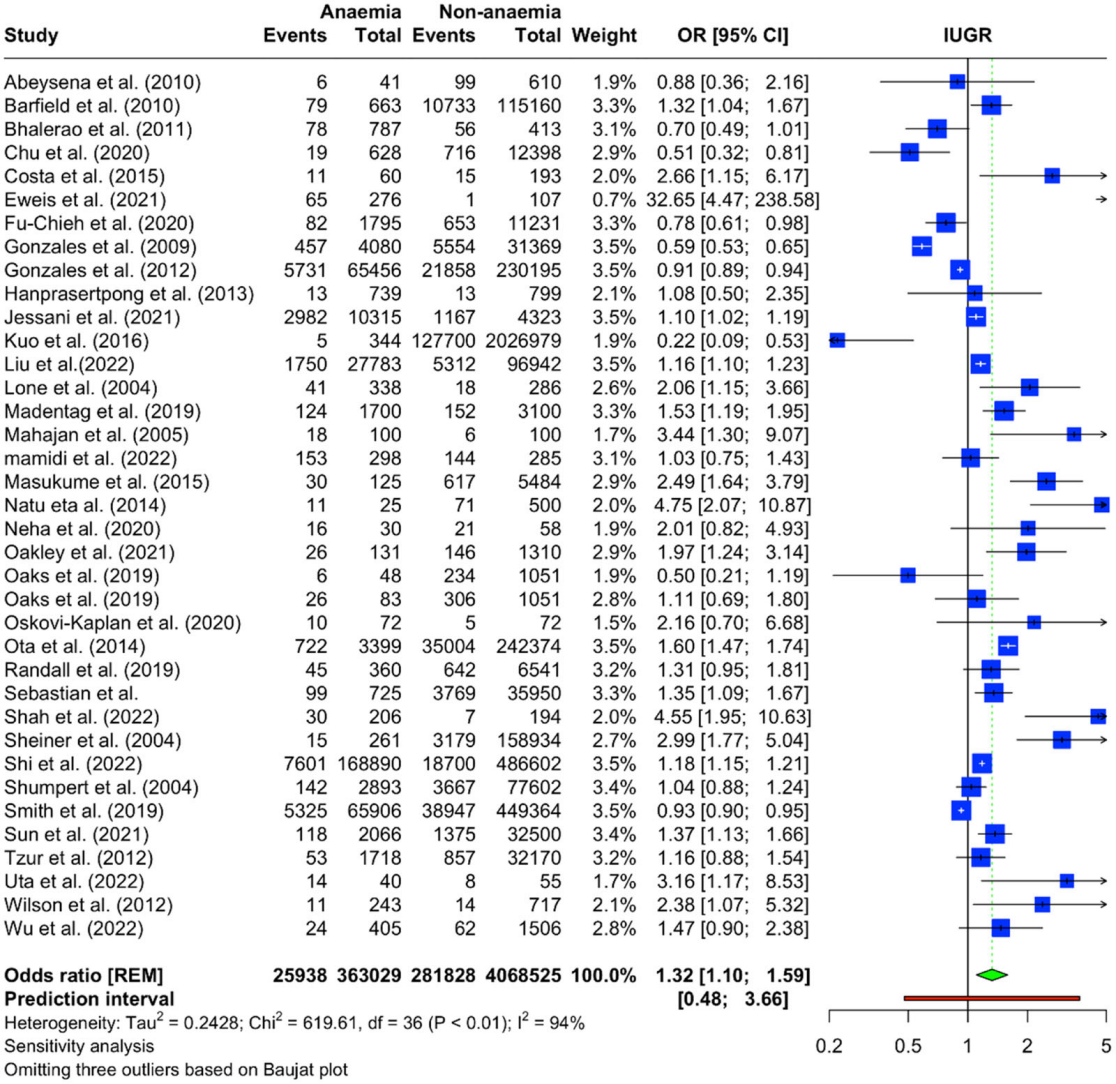
Re‐analysis after removing outliers.

Sensitivity analyses were conducted by sequentially omitting each included study, and the resulting changes in the pooled proportion/risk ratio were found to be non‐significant, suggesting that the results of the meta‐analysis were stable (Supporting Information S1: Figure S1). The Baujat plot identified three outliers (Supporting Information S1: Figure S2). A reanalysis was performed after removing these outliers (Figure 7). Following this adjustment, the association between IUGR and maternal anaemia remained significant with an OR of 1.32 (95% CI: 1.10–1.59).

### 3.7 Meta‐Regression

Meta‐regression was performed to assess the effect of sample size and age on the results (Supporting Information S1: Figures S3 and S4). However, the meta‐regression revealed no significant effect of age (*p* = 0.151) or sample size (*p* = 0.954) on the outcomes.

### 3.8 Publication Bias

**Figure 8:**
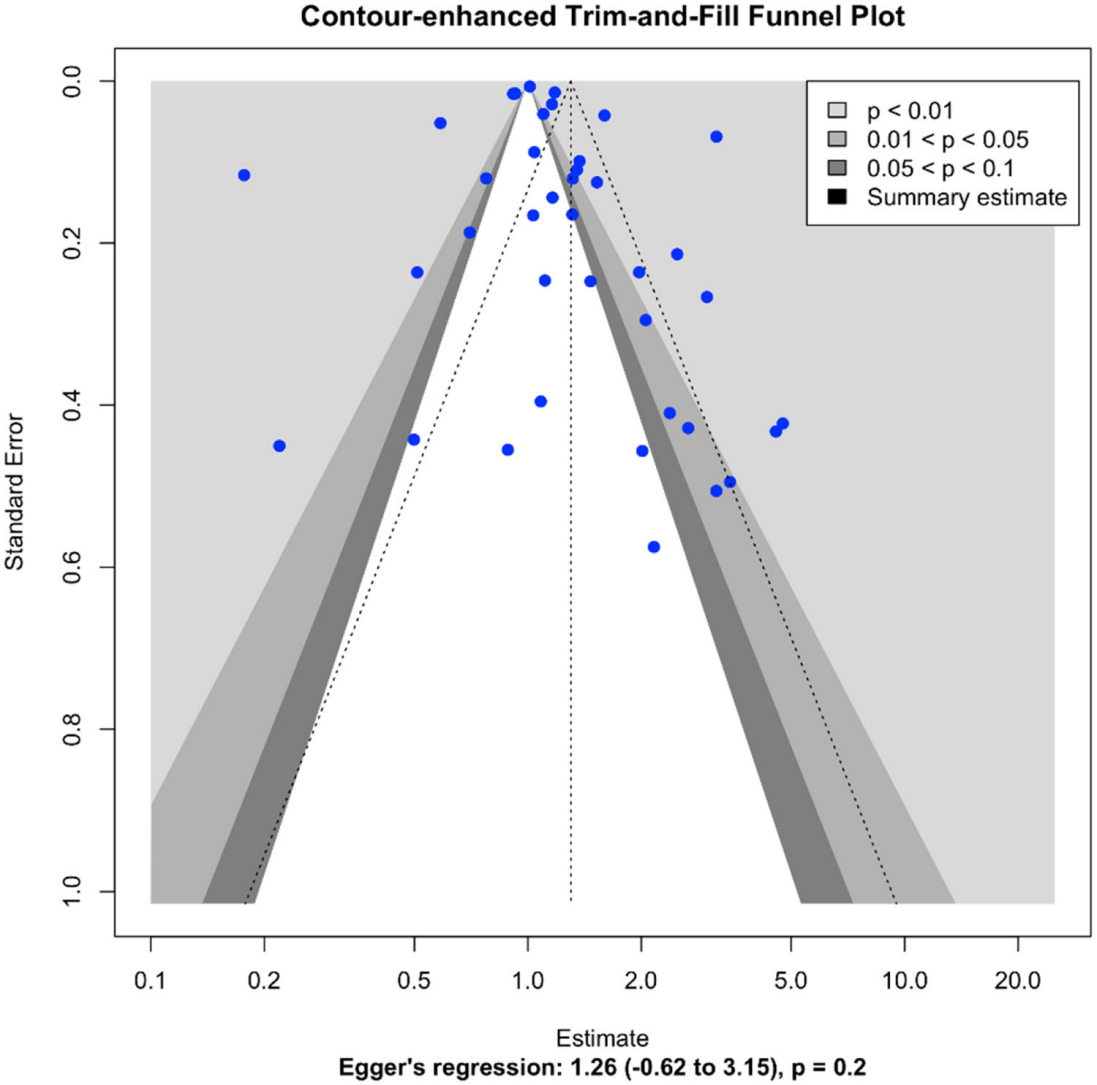
Funnel plot assessing the presence of publication bias.

The contour‐enhanced trim‐and‐fill funnel plot and Egger's regression test were conducted to assess publication bias. The slight asymmetry of the plot was observed, but Egger's regression test indicates no significant evidence of publication bias (intercept = 1.26, 95% CI: −0.62 to 3.15, *p* = 0.2). This indicates less possibility for the presence of publication bias (Figure 8).

## 4 Discussion

This systematic review and meta‐analysis consolidate evidence on the association between maternal anaemia and the risk of IUGR, an important adverse outcome of pregnancy. Our analysis included 38 studies and revealed that anaemia in pregnant women is associated with 30% increased odds of IUGR compared to non‐anaemic women. The substantial heterogeneity observed (*I*² = 97%) likely reflects varied diagnostic criteria for anaemia, differing severities of anaemia and diverse geographic and demographic factors among the included studies. Subgroup analysis based on the severity of anaemia did not show any significant associations. For severe anaemia, the effect size suggested a potentially higher risk, even though it was statistically nonsignificant. Only a few studies categorized the severity of anaemia. Therefore, the evidence regarding different levels of anaemia and IUGR is limited.

The adverse birth outcomes associated with anaemia during pregnancy are evident from previous systematic reviews as well. For instance, a previous meta‐analysis demonstrated a significant relationship between maternal anaemia and low birth weight in infants during the first trimester of pregnancy (Rahmati et al. 2017). Another meta‐analysis found that maternal anaemia was associated with a significantly higher risk of adverse birth outcomes (Rahman, Khan, and Rahman 2020). Specifically, it increased the odds of low birth weight by 90% (OR: 1.90), nearly doubled the risk of preterm birth (OR: 1.96) and elevated the likelihood of perinatal mortality nearly threefold (OR: 2.90). Conversely, the authors reported that the relationships between maternal anaemia and other outcomes such as neonatal mortality, miscarriage, pre‐eclampsia and caesarean delivery were not statistically significant (Rahman, Khan, and Rahman 2020).

The findings of this study, along with previous research, have significant clinical implications for the management of maternal health, particularly, in the context of prenatal care. The association between maternal anaemia and the increased risk of IUGR underscores the necessity for early diagnosis and effective management of anaemia during pregnancy. Given the diverse causes of anaemia, which include iron deficiency, vitamin B12 and folate deficiencies and genetic conditions such as thalassaemia and sickle cell disease, tailored treatment plans are essential (Das et al. 2023). For example, supplementation with iron and folate could be, particularly, impactful in populations where dietary intake is insufficient (Yuan et al. 2022). Furthermore, the management plan should also include monitoring and potentially treating other risk factors that can exacerbate anaemia's effects on foetal growth, such as malnutrition and chronic infections. Selecting the appropriate treatment is also crucial. A recent systematic review has shown that findings from meta‐regression analysis indicate that IV iron is more likely to reduce maternal complications by 21% compared to oral iron (Pandey et al. 2024). IV iron increases haemoglobin more significantly and at a faster pace than oral iron. The authors concluded that intravenous iron is more likely to avert adverse maternal outcomes and adverse reactions (Pandey et al. 2024). Intravenous iron should therefore be considered in clinical settings where rapid correction of anaemia is required and in cases where oral iron therapy fails or is contraindicated.

The geographic distribution of the studies included in this meta‐analysis provides valuable insight into how regional variations may influence both the prevalence of anaemia in pregnancy and its association with IUGR. The studies span a range of geographic contexts, from high‐income countries like the United States, the United Kingdom and Australia, to low‐ and middle‐income countries such as India, Ghana and Peru. These differences are important to consider, as they reflect disparities in healthcare infrastructure, nutritional status and access to prenatal care, which may in turn affect the maternal and foetal outcomes related to anaemia. In low‐ and middle‐income countries, the higher prevalence of anaemia is often linked to factors such as malnutrition, limited access to iron supplementation and suboptimal healthcare services, all of which can exacerbate the risk of adverse pregnancy outcomes like IUGR (Kinyoki et al. 2021). For instance, in parts of sub‐Saharan Africa and South Asia, the prevalence of iron deficiency anaemia is significantly higher, often due to food insecurity, infectious diseases and inadequate healthcare infrastructure (Tesema et al. 2021). On the other hand, high‐income countries generally have more robust healthcare systems, better access to prenatal care and more widespread use of nutritional supplementation during pregnancy, which may mitigate the impact of anaemia on foetal growth. However, disparities still exist within these countries, particularly, in underserved communities or among certain ethnic groups with higher rates of genetic haemoglobinopathies such as sickle cell anaemia or thalassaemia.

There is a need for large‐scale, longitudinal studies that can track the progression of anaemia throughout pregnancy and its direct impacts on foetal development. Such studies should aim to establish a clear causal relationship and identify critical windows during pregnancy when intervention is most beneficial. Research should explore the differential impacts of various types of anaemia, such as iron deficiency versus anaemia caused by chronic diseases or genetic disorders. Understanding these distinctions is crucial for developing more targeted therapies. Additionally, studies evaluating the efficacy of different iron supplementation regimens (oral vs. intravenous) in diverse populations could provide much‐needed data to guide clinical practices, particularly, in low‐resource settings. Another important area for investigation is the interplay between maternal anaemia and other maternal risk factors, such as maternal malnutrition, smoking or pre‐existing chronic conditions. Multifactorial studies that consider these complex interactions may reveal synergistic or protective effects that single‐factor analyses might miss. Moreover, the development and validation of innovative treatment modalities or supplementation programs that are cost‐effective, safe and culturally acceptable across different global regions would also be beneficial. There is a compelling need to assess the long‐term outcomes of infants born with IUGR associated with maternal anaemia to understand the full scope of developmental challenges these individuals might face.

This study benefits from a rigorous methodological framework following PRISMA guidelines and robust statistical analysis. Our team included multidisciplinary researchers. However, there are limitations. The high heterogeneity among included studies suggests variability in study designs, populations, anaemia definitions and assessment methods, which could affect the generalizability of the findings. Only a few studies reported different severity levels of anaemia, so the analysis of the dose–response relationship is not robust. Additionally, we only included articles published in English, which might have excluded relevant studies in other languages. Furthermore, there could be an interplay between maternal anaemia and other risk factors (e.g., maternal malnutrition, infection status) that could confound or mediate the relationship with IUGR. While the funnel plot showed slight asymmetry, Egger's test did not indicate significant publication bias. However, the presence of asymmetry may suggest that smaller studies with less significant findings could be underrepresented. This could potentially lead to an overestimation of the overall effect size. We acknowledge this limitation and emphasize that while the results are robust, this slight asymmetry should be considered when interpreting the findings. Future research should aim to include more small‐scale studies to reduce potential bias.

## 5 Conclusion

Maternal anaemia is significantly associated with an increased risk of IUGR. More studies are required to analyze the different severities of anaemia and IUGR. Our analysis reveals the critical need for proactive management of maternal anaemia within antenatal care frameworks to improve pregnancy outcomes. Addressing this global health issue requires coordinated efforts involving enhanced screening, better nutritional and healthcare interventions, and comprehensive educational programs to raise awareness among healthcare providers and the community about the implications of maternal anaemia.

## Author Contributions

**Rajeev Jayalakshmi:** data curation, formal analysis, supervision. **Shilpa Gaidhane:** conceptualization, review and editing of drafts, methodology. **Suhas Ballal:** data curation, formal analysis. **Sanjay Kumar:** data curation, formal analysis. **Mahakshit Bhat:** data curation, formal analysis. **Shilpa Sharma:** investigation. **M. Ravi Kumar:** investigation. **Sarvesh Rustagi:** project administration, resources. **Mahalaqua Nazli Khatib:** project administration, resources. **Nishant Rai:** software, validation. **Sanjit Sah:** software, validation. **Sorabh Lakhanpal:** data curation, formal analysis, project administration, resources. **Hashem Abu Serhan:** software, validation. **Ganesh Bushi:** writing–original draft, writing–review and editing. **Muhammed Shabil:** conceptualization, review and editing of drafts, methodology.

## Ethics Statement

The authors have nothing to report.

## Conflicts of Interest

The authors declare no conflicts of interest.

## Supporting information

Supporting information.

## Data Availability

The data are with the authors and available on request.
