# Supplementary material for: The Effect of Maternal Haemoglobinopathies and Iron Deficiency Anaemia on Foetal Growth Restriction: A Systematic Review and Meta‐Analysis
Source: Matern Child Nutr. 2025 Apr 15;21(3):e13787. doi: 10.1111/mcn.13787 (PMC12150161; doi:10.1111/mcn.13787)
Supplement: Supplementary file 1 — Supporting information. [file MCN-21-e13787-s001.docx]

**Supplementary Materials**

**Table S1.** PRISMA Checklist

**Table S2.** The adjusted search terms as per searched electronic databases

**Table S3.** Newcastle-Ottawa Scale for the quality assessment of studies

**Figure S1**: Sensitivity analysis (Leave-one-out)

**Figure S2**: Baujat plot

**Figure S3:** Bubble plot of meta-regression result based on sample size

**Figure S4:** Bubble plot of meta-regression result based on age

## **Table S1.** PRISMA Checklist

| **Section and Topic** | **Item #** | **Checklist item (Prevalence of kidney diseases among the dengue patients: A systematic review and meta-analysis)** | **Location where item is reported** |
| --- | --- | --- | --- |
| **TITLE** | | |  |
| Title | 1 | Identify the report as a systematic review. | 1 |
| **ABSTRACT** | | |  |
| Abstract | 2 | See the PRISMA 2020 for Abstracts checklist. (made as per the Journal guidelines) | 2 |
| **INTRODUCTION** | | |  |
| Rationale | 3 | Describe the rationale for the review in the context of existing knowledge. | 3 |
| Objectives | 4 | Provide an explicit statement of the objective(s) or question(s) the review addresses. | 3 |
| **METHODS** | | |  |
| Eligibility criteria | 5 | Specify the inclusion and exclusion criteria for the review and how studies were grouped for the syntheses. | 4 |
| Information sources | 6 | Specify all databases, registers, websites, organisations, reference lists and other sources searched or consulted to identify studies. Specify the date when each source was last searched or consulted. | 4, Table S3 |
| Search strategy | 7 | Present the full search strategies for all databases, registers and websites, including any filters and limits used. | Table S3 |
| Selection process | 8 | Specify the methods used to decide whether a study met the inclusion criteria of the review, including how many reviewers screened each record and each report retrieved, whether they worked independently, and if applicable, details of automation tools used in the process. | 4 |
| Data collection process | 9 | Specify the methods used to collect data from reports, including how many reviewers collected data from each report, whether they worked independently, any processes for obtaining or confirming data from study investigators, and if applicable, details of automation tools used in the process. | 4 |
| Data items | 10a | List and define all outcomes for which data were sought. Specify whether all results that were compatible with each outcome domain in each study were sought (e.g., for all measures, time points, analyses), and if not, the methods used to decide which results to collect. | 3 |
|  | 10b | List and define all other variables for which data were sought (e.g., participant and intervention characteristics, funding sources). Describe any assumptions made about any missing or unclear information. | 4, Table S4 |
| Study risk of bias assessment | 11 | Specify the methods used to assess risk of bias in the included studies, including details of the tool(s) used, how many reviewers assessed each study and whether they worked independently, and if applicable, details of automation tools used in the process. | Table S4 |
| Effect measures | 12 | Specify for each outcome the effect measure(s) (e.g. risk ratio, mean difference) used in the synthesis or presentation of results. | 5 |
| Synthesis methods | 13a | Describe the processes used to decide which studies were eligible for each synthesis (e.g. tabulating the study intervention characteristics and comparing against the planned groups for each synthesis (item #5)). | 4,5 |
|  | 13b | Describe any methods required to prepare the data for presentation or synthesis, such as handling of missing summary statistics, or data conversions. | NA |
|  | 13c | Describe any methods used to tabulate or visually display results of individual studies and syntheses. |  |
|  | 13d | Describe any methods used to synthesize results and provide a rationale for the choice(s). If meta-analysis was performed, describe the model(s), method(s) to identify the presence and extent of statistical heterogeneity, and software package(s) used. | 4.5 |
|  | 13e | Describe any methods used to explore possible causes of heterogeneity among study results (e.g. subgroup analysis, meta-regression). | 5 |
|  | 13f | Describe any sensitivity analyses conducted to assess robustness of the synthesized results. | 6, Figure 3,4 |
| Reporting bias assessment | 14 | Describe any methods used to assess risk of bias due to missing results in a synthesis (arising from reporting biases). | 5 |
| Certainty assessment | 15 | Describe any methods used to assess certainty (or confidence) in the body of evidence for an outcome. | NA |
| **RESULTS** | | |  |
| Study selection | 16a | Describe the results of the search and selection process, from the number of records identified in the search to the number of studies included in the review, ideally using a flow diagram. | Table S2 |
|  | 16b | Cite studies that might appear to meet the inclusion criteria, but which were excluded, and explain why they were excluded. | NA |
| Study characteristics | 17 | Cite each included study and present its characteristics. | 4,5 Table 1 |
| Risk of bias in studies | 18 | Present assessments of risk of bias for each included study. | Table S4 |
| Results of individual studies | 19 | For all outcomes, present, for each study: (a) summary statistics for each group (where appropriate) and (b) an effect estimate and its precision (e.g. confidence/credible interval), ideally using structured tables or plots. | Table 1, Figure 2 |
| Results of syntheses | 20a | For each synthesis, briefly summarise the characteristics and risk of bias among contributing studies. | 4 |
|  | 20b | Present results of all statistical syntheses conducted. If meta-analysis was done, present for each the summary estimate and its precision (e.g. confidence/credible interval) and measures of statistical heterogeneity. If comparing groups, describe the direction of the effect. | 5,4 Figure 2 |
|  | 20c | Present results of all investigations of possible causes of heterogeneity among study results. | 5, Figure 3 |
|  | 20d | Present results of all sensitivity analyses conducted to assess the robustness of the synthesized results. | Figure 3 |
| Reporting biases | 21 | Present assessments of risk of bias due to missing results (arising from reporting biases) for each synthesis assessed. | NA |
| Certainty of evidence | 22 | Present assessments of certainty (or confidence) in the body of evidence for each outcome assessed. | NA |
| **DISCUSSION** | | |  |
| Discussion | 23a | Provide a general interpretation of the results in the context of other evidence. | 5,6, 7 |
|  | 23b | Discuss any limitations of the evidence included in the review. | 7 |
|  | 23c | Discuss any limitations of the review processes used. | 7 |
|  | 23d | Discuss implications of the results for practice, policy, and future research. | 7 |
| **OTHER INFORMATION** | | |  |
| Registration and protocol | 24a | Provide registration information for the review, including register name and registration number, or state that the review was not registered. | 3 |
|  | 24b | Indicate where the review protocol can be accessed, or state that a protocol was not prepared. | 3 |
|  | 24c | Describe and explain any amendments to information provided at registration or in the protocol. | NA |
| Support | 25 | Describe sources of financial or non-financial support for the review, and the role of the funders or sponsors in the review. | 8 |
| Competing interests | 26 | Declare any competing interests of review authors. | 7 |
| Availability of data, code and other materials | 27 | Report which of the following are publicly available and where they can be found: template data collection forms; data extracted from included studies; data used for all analyses; analytic code; any other materials used in the review. | Supplementary Materials |

**Table S2.** The adjusted search terms used to extract literature from electronic databases.

| Database | No | Search Query | Results |
| --- | --- | --- | --- |
|  | |  | |
| PubMed | **#1** | ((((Anemia[Mesh]) OR ("Iron deficiency"[tiab])) OR ("Anemia, Iron-Deficiency"[Mesh])) OR (Hemoglobin[tiab])) OR (Anaemia[ti]) | 78,177 |
|  | **#2** | ((("pregnant women"[Mesh]) OR (pregnan*[tiab])) OR (maternal[tiab])) OR (mother*[tiab]) | 381,382 |
|  | **#3** | ((((("Growth Restrictions"[tiab])) OR ("small for gestational age"[tiab])) OR ("intrauterine growth restriction"[tiab])) OR (IUGR[ti])) | 11,665 |
|  | **#4** | **#1 AND #2 AND #3** | **336** |
| Cochrane | **#1** | (anemia):ti,ab,kw OR (Iron deficiency):ti,ab,kw OR (Anaemia):ti,ab,kw OR (hemoglobin):ti,ab,kw | 61,775 |
|  | **#2** | (pregnant woman):ti,ab,kw OR (pregnan*):ti,ab,kw OR (maternal):ti,ab,kw OR (mother*):ti,ab,kw | 100,791 |
|  | **#3** | (Growth Restrictions):ti,ab,kw OR (small for gestational age):ti,ab,kw OR (intrauterine growth restriction): ti,ab,kw OR (IUGR):ti,ab,kw | 2,992 |
|  | **#4** | #1 AND #2 AND #3 **(Cochrane reviews 6 + 272 trials)** | **6** |
| EMBASE | **#1** | ((((Anemia/exp) OR ('Iron deficiency':ti,ab)) OR ('Anemia, Iron-Deficiency'/exp)) OR (Hemoglobin:ti,ab)) OR (Anaemia:ti) | 251,866 |
|  | **#2** | ((('pregnant woman'/exp) OR (pregnan*:ti,ab)) OR (maternal:ti,ab)) OR (mother*:ti,ab) | 562,655 |
|  | **#3** | ('growth restrictions':ti,ab OR 'small for gestational age':ti,ab OR 'intrauterine growth restriction':ti,ab OR iugr:ti) | 17,261 |
|  | **#4** | #1 AND #2 AND #3 | **890** |
| WOS  advanced | **#1** | (((TS=(Anemia)) OR TS=("Iron deficiency")) OR TS=(Hemoglobin)) OR TS=(Anaemia) | 204,655 |
|  | **#2** | (((ALL="pregnant woman") OR ((TI=pregnan* OR AB=pregnan*))) OR ((TI=maternal OR AB=maternal))) OR ((TI=mother* OR AB=mother*)) | 454,355 |
|  | **#3** | ((((((TI="Growth Restrictions" OR AB="Growth Restrictions"))) OR ((TI="small for gestational age" OR AB="small for gestational age"))) OR ((TI="intrauterine growth restriction" OR AB="intrauterine growth restriction"))) OR (TI=IUGR)) | 14,655 |
|  | **#4** | #1 AND #2 AND #3 | **458** |

**Total:** 1690

**Table S4.** Newcastle-Ottawa Scale tool for the quality assessment of studies

| **STUDY** | **SELECTION (max 4 points)** | | | | **COMPARABILITY (max 2 points)** | **OUTCOME (max 3 points)** | | | **SCORE (out of 9)** |
| --- | --- | --- | --- | --- | --- | --- | --- | --- | --- |
|  | Representativeness | Selection | Ascertainment | Demonstration of the outcome of interest was not present at start of study | Comparability the basis of the design or analysis | Assessment of outcome | Was follow-up long enough for outcomes to occur? | Adequacy of the follow-up |  |
| Abeysena et al. (2010) (1) | 1 | 1 | 1 | 0 | 1 | 1 | 1 | 0 | 6 |
| Barfield et al. (2010) (2) | 1 | 1 | 1 | 1 | 1 | 1 | 0 | 1 | 7 |
| Bhalerao et al. (2011) (3) | 1 | 1 | 1 | 1 | 1 | 1 | 0 | 0 | 6 |
| Chu et al. (2020) (4) | 1 | 1 | 1 | 0 | 1 | 1 | 0 | 1 | 7 |
| Costa et al. (2015) (5) | 1 | 1 | 1 | 1 | 2 | 1 | 1 | 1 | 8 |
| Eweis et al. (2021) (6) | 1 | 1 | 1 | 1 | 1 | 1 | 1 | 0 | 7 |
| Fu-Chieh et al. (2020) (7) | 1 | 1 | 1 | 1 | 2 | 1 | 1 | 0 | 8 |
| Gonzales et al. (2009) (8) | 1 | 1 | 1 | 1 | 1 | 1 | 1 | 0 | 7 |
| Gonzales et al. (2012) (9) | 1 | 1 | 1 | 0 | 1 | 1 | 1 | 0 | 6 |
| Hanprasertpong et al. (2013) (10) | 1 | 1 | 1 | 1 | 1 | 1 | 0 | 1 | 7 |
| Jasim et al. (2020) (11) | 1 | 1 | 1 | 1 | 1 | 1 | 1 | 1 | 7 |
| Jessani et al. (2021) (12) | 1 | 1 | 1 | 1 | 2 | 1 | 1 | 0 | 8 |
| Kuo et al. (2016) (13) | 1 | 1 | 1 | 1 | 1 | 1 | 1 | 0 | 7 |
| Lin et al. (2010) (14) | 1 | 1 | 1 | 1 | 1 | 1 | 0 | 0 | 5 |
| Liu et al. (2022) (15) | 1 | 1 | 1 | 1 | 1 | 1 | 0 | 0 | 6 |
| Lone et al. (2004) (16) | 1 | 1 | 1 | 0 | 1 | 1 | 0 | 1 | 7 |
| Madentag et al. (2019) (17) | 1 | 1 | 1 | 1 | 2 | 1 | 1 | 1 | 8 |
| Mahajan et al. (2005) (18) | 1 | 1 | 1 | 1 | 1 | 1 | 1 | 0 | 7 |
| Mamidi et al. (2022) (19) | 1 | 1 | 1 | 1 | 2 | 1 | 1 | 0 | 8 |
| Masukume et al. (2015) (20) | 1 | 1 | 1 | 0 | 1 | 1 | 1 | 0 | 6 |
| Natu eta al. (2014) (21) | 1 | 1 | 1 | 1 | 1 | 1 | 0 | 1 | 7 |
| Neha et al. (2020) (22) | 1 | 1 | 1 | 1 | 1 | 1 | 0 | 0 | 6 |
| Oakley et al. (2021) (23) | 1 | 1 | 1 | 0 | 1 | 1 | 0 | 1 | 7 |
| Oaks et al. (2019) (24) | 1 | 1 | 1 | 1 | 2 | 1 | 1 | 1 | 5 |
| Oskovi-Kaplan et al. (2020) (25) | 1 | 1 | 1 | 1 | 1 | 1 | 1 | 0 | 7 |
| Ota et al. (2014) (26) | 1 | 1 | 1 | 0 | 1 | 0 | 1 | 0 | 6 |
| Randall et al. (2019) (27) | 1 | 1 | 1 | 1 | 1 | 1 | 0 | 1 | 7 |
| Sebastian et al. (2015) (28) | 1 | 1 | 1 | 1 | 1 | 1 | 0 | 0 | 6 |
| Shah et al. (2022) (29) | 1 | 1 | 1 | 1 | 2 | 1 | 1 | 1 | 8 |
| Sheiner et al. (2004) (30) | 1 | 1 | 1 | 1 | 1 | 1 | 1 | 0 | 7 |
| Shi et al. (2022) (31) | 1 | 1 | 1 | 1 | 2 | 1 | 1 | 0 | 8 |
| Shumpert et al. (2004) (32) | 1 | 1 | 1 | 1 | 1 | 1 | 1 | 0 | 7 |
| Smith et al. (2019) (33) | 1 | 1 | 1 | 0 | 1 | 1 | 1 | 0 | 6 |
| Sun et al. (2021) (34) | 1 | 1 | 1 | 1 | 1 | 1 | 0 | 1 | 7 |
| Tzur et al. (2012) (35) | 1 | 1 | 1 | 1 | 1 | 1 | 1 | 1 | 7 |
| Uta et al. (2022) (36) | 1 | 1 | 1 | 1 | 2 | 1 | 1 | 0 | 8 |
| Wilson et al. (2012) (37) | 1 | 1 | 1 | 1 | 1 | 1 | 1 | 0 | 7 |
| Wu et al. (2022) (38) | 1 | 1 | 1 | 1 | 2 | 1 | 1 | 1 | 8 |


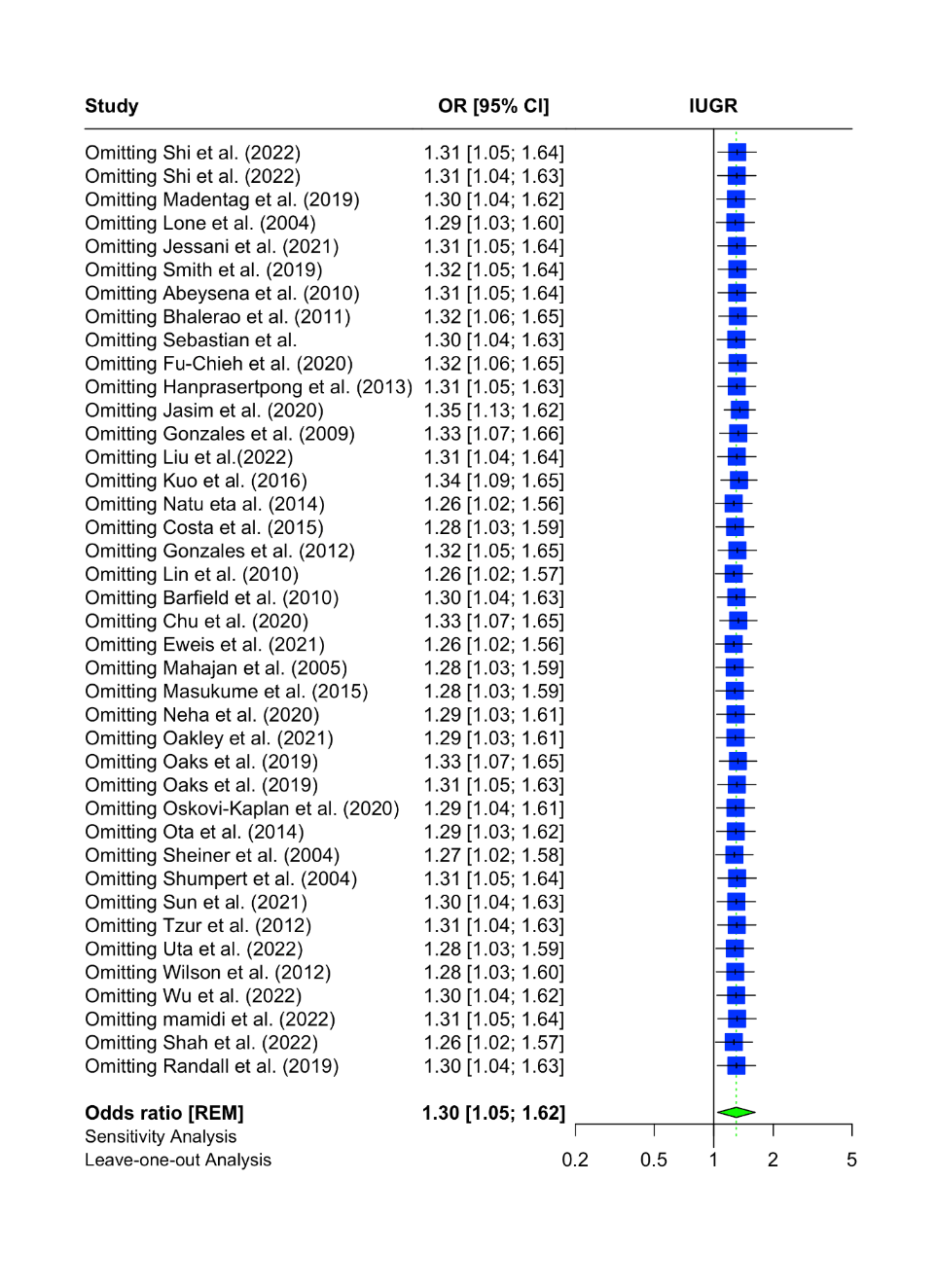


**Figure S1**: Sensitivity analysis (Leave-one-out)


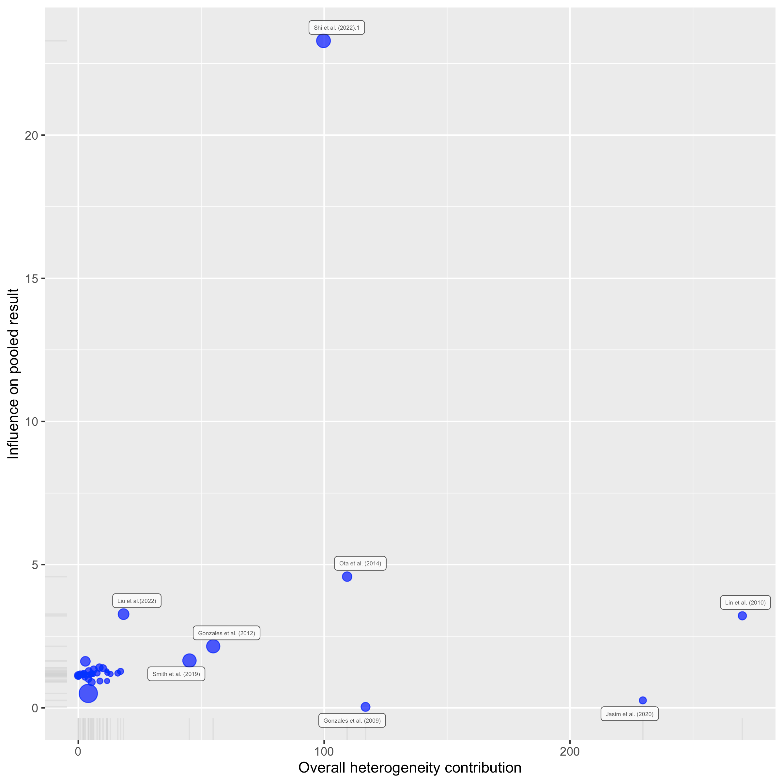


**Figure S2**: Baujat plot


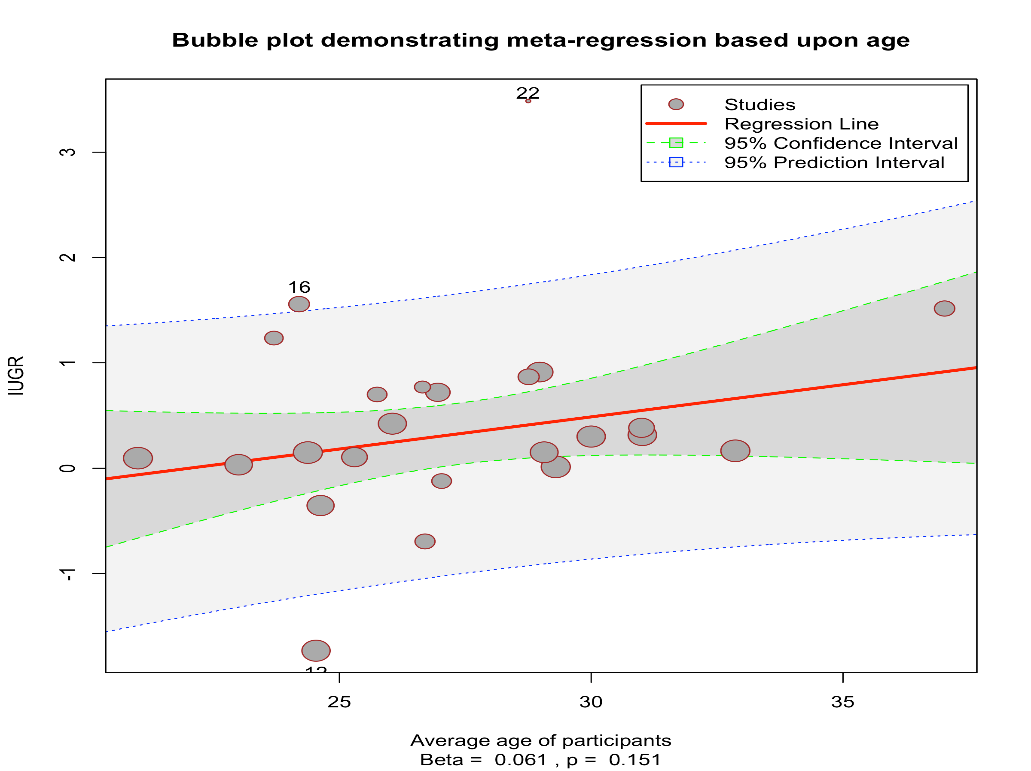


**Figure S3:** Bubble plot of meta-regression result based on sample size


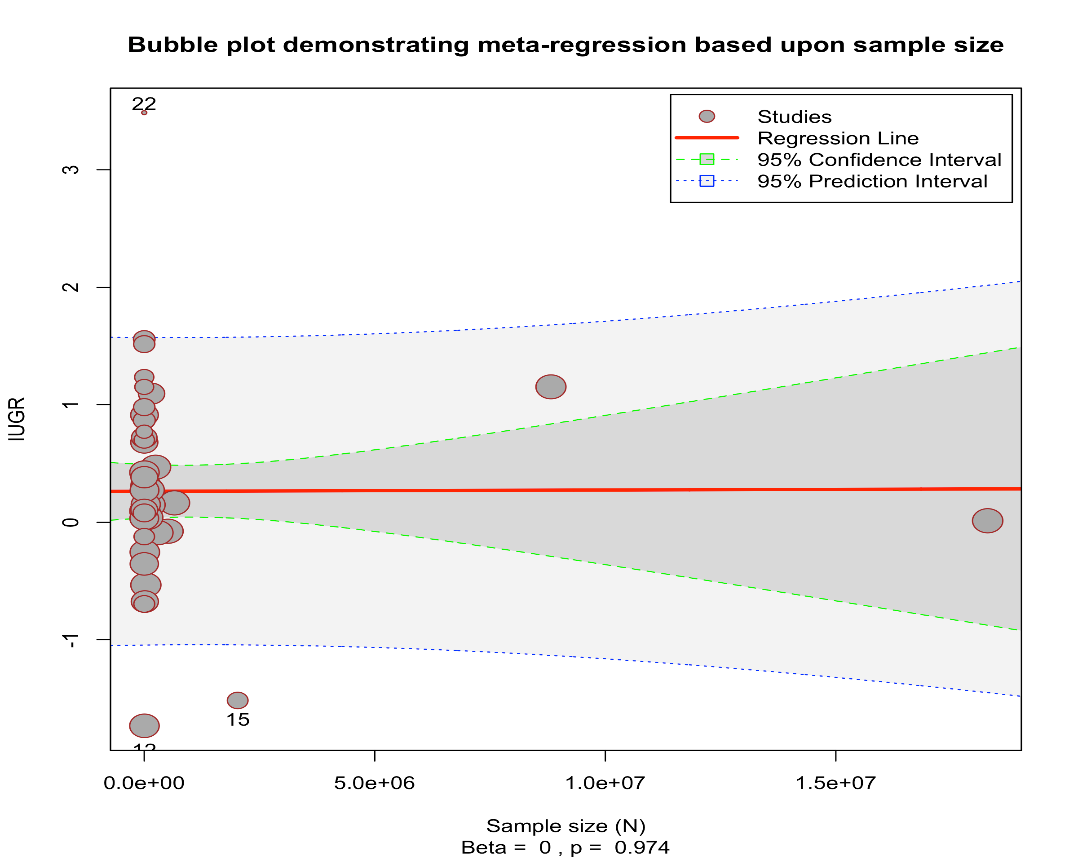


**Figure S4:** Bubble plot of meta-regression result based on age

**Bibliography**

1. Abeysena C, Jayawardana P, De A. SENEVIRATNE R. Maternal haemoglobin level at booking visit and its effect on adverse pregnancy outcome. Australian and New Zealand Journal of Obstetrics and Gynaecology. 2010;50(5):423-7.

2. Barfield WD, Barradas DT, Manning SE, Kotelchuck M, Shapiro-Mendoza CK. Sickle cell disease and pregnancy outcomes: women of African descent. American journal of preventive medicine. 2010;38(4):S542-S9.

3. Joshi S, Ghike S, Bhalerao A, Kawthalkar A. Anemia during pregnancy: Most preventable yet Most Prevalent. Journal of South Asian Federation of Obstetrics and Gynaecology. 2013;3(2):75-7.

4. Chu F-C, Shao SS-W, Lo L-M, Hung T-H. Association between maternal anemia at admission for delivery and adverse perinatal outcomes. Journal of the Chinese Medical Association. 2020;83(4):402-7.

5. Costa VMF, Viana MB, Aguiar RALP. Pregnancy in patients with sickle cell disease: maternal and perinatal outcomes. The Journal of Maternal-Fetal & Neonatal Medicine. 2015;28(6):685-9.

6. Eweis M, Farid EZ, El-Malky N, Abdel-Rasheed M, Salem S, Shawky S. Prevalence and determinants of anemia during the third trimester of pregnancy. Clinical Nutrition ESPEN. 2021;44:194-9.

7. Chu F-C, Shen-Wen Shao S, Lo L-M, Hsieh Ts-Ta, Hung T-H. Association between maternal anemia at admission for delivery and adverse perinatal outcomes. Journal of the Chinese Medical Association. 2020;83(4):402-7.

8. Gonzales GF, Steenland K, Tapia V. Maternal hemoglobin level and fetal outcome at low and high altitudes. Am J Physiol Regul Integr Comp Physiol. 2009;297(5):R1477-85.

9. Gonzales GF, Tapia V, Gasco M, Carrillo CE. Maternal hemoglobin concentration and adverse pregnancy outcomes at low and moderate altitudes in Peru. The Journal of Maternal-Fetal & Neonatal Medicine. 2012;25(7):1105-10.

10. Hanprasertpong T, Hanprasertpong J. Pregnancy outcomes in Southeast Asian migrant workers at Southern Thailand. Journal of Obstetrics and Gynaecology. 2015;35(6):565-9.

11. Jasim SK, Al-Momen H, Al-Asadi F. Maternal anemia prevalence and subsequent neonatal complications in Iraq. Open Access Macedonian Journal of Medical Sciences. 2020;8(B):71-5.

12. Jessani S, Saleem S, Hoffman MK, Goudar SS, Derman RJ, Moore JL, et al. Association of haemoglobin levels in the first trimester and at 26–30 weeks with fetal and neonatal outcomes: a secondary analysis of the Global Network for Women's and Children's Health's ASPIRIN Trial. BJOG: An International Journal of Obstetrics & Gynaecology. 2021;128(9):1487-96.

13. Kuo K, Caughey AB. Contemporary outcomes of sickle cell disease in pregnancy. American journal of obstetrics and gynecology. 2016;215(4):505. e1-. e5.

14. Lin H-C, Chiu C-CJ, Chen S-F, Lou H-Y, Chiu W-T, Chen Y-H. Ulcerative colitis and pregnancy outcomes in an Asian population. Official journal of the American College of Gastroenterology| ACG. 2010;105(2):387-94.

15. Liu X, An H, Li N, Li Z, Zhang Y, Zhang L, et al. Preconception Hemoglobin Concentration and Risk of Low Birth Weight and Small-for-Gestational-Age: A Large Prospective Cohort Study in China. Nutrients. 2022;14(2).

16. Lone F, Qureshi R, Emmanuel F. Maternal anaemia and its impact on perinatal outcome in a tertiary care hospital in Pakistan. EMHJ-Eastern Mediterranean Health Journal, 10 (6), 801-807, 2004. 2004.

17. Col Madendag I, Eraslan Sahin M, Madendag Y, Sahin E, Demir MB, Acmaz B, et al. The Effect of Iron Deficiency Anemia Early in the Third Trimester on Small for Gestational Age and Birth Weight: A Retrospective Cohort Study on Iron Deficiency Anemia and Fetal Weight. Biomed Res Int. 2019;2019:7613868.

18. Mahajan SD, Aalinkeel R, Singh S, Shah P, Gupta N, Kochupillai N. Thyroid hormone dysregulation in intrauterine growth retardation associated with maternal malnutrition and/or anemia. Horm Metab Res. 2005;37(10):633-40.

19. Mamidi RS, Banjara SK, Manchala S, Babu CK, Geddam JJB, Boiroju NK, et al. Maternal Nutrition, Body Composition and Gestational Weight Gain on Low Birth Weight and Small for Gestational Age—A Cohort Study in an Indian Urban Slum. Children. 2022;9(10):1460.

20. Masukume G, Khashan AS, Kenny LC, Baker PN, Nelson G. Risk factors and birth outcomes of anaemia in early pregnancy in a nulliparous cohort. PLoS One. 2015;10(4):e0122729.

21. Natu N, Khandelwal S, Kumar R, Dave A. Maternal and perinatal outcome of women with sickle cell disease of a tribal population in Central India. Hemoglobin. 2014;38(2):91-4.

22. Rao SS, Baliga BS, Mithra P, Manjrekar P, Kamath N. Influencing variables for fetal growth in malnourished mothers: A nested case-control study. Clinical Epidemiology and Global Health. 2020;8(2):581-5.

23. Oakley LL, Mitchell S, von Rege I, Hadebe R, Howard J, Robinson SE, Oteng‐Ntim E. Perinatal outcomes in women with sickle cell disease: a matched cohort study from London, UK. British Journal of Haematology. 2022;196(4):1069-75.

24. Oaks BM, Jorgensen JM, Baldiviez LM, Adu-Afarwuah S, Maleta K, Okronipa H, et al. Prenatal iron deficiency and replete iron status are associated with adverse birth outcomes, but associations differ in Ghana and Malawi. The Journal of nutrition. 2019;149(3):513-21.

25. Oskovi-Kaplan ZA, Kilickiran H, Buyuk GN, Ozyer S, Keskin HL, Engin-Ustun Y. Comparison of the maternal and neonatal outcomes of pregnant women whose anemia was not corrected before delivery and pregnant women who were treated with intravenous iron in the third trimester. Archives of Gynecology and Obstetrics. 2021;303:715-9.

26. Ota E, Ganchimeg T, Morisaki N, Vogel JP, Pileggi C, Ortiz-Panozo E, et al. Risk factors and adverse perinatal outcomes among term and preterm infants born small-for-gestational-age: secondary analyses of the WHO Multi-Country Survey on Maternal and Newborn Health. PLoS One. 2014;9(8):e105155.

27. Randall DA, Patterson JA, Gallimore F, Morris JM, Simpson JM, McGee TM, et al. Haemoglobin trajectories during pregnancy and associated outcomes using pooled maternity and hospitalization data from two tertiary hospitals. Vox Sanguinis. 2019;114(8):842-52.

28. Sebastian T, Yadav B, Jeyaseelan L, Vijayaselvi R, Jose R. Small for gestational age births among South Indian women: temporal trend and risk factors from 1996 to 2010. BMC pregnancy and childbirth. 2015;15:1-10.

29. Shah T, Khaskheli MS, Ansari S, Lakhan H, Shaikh F, Zardari AA, et al. Gestational Anemia and its effects on neonatal outcome, in the population of Hyderabad, Sindh, Pakistan. Saudi Journal of Biological Sciences. 2022;29(1):83-7.

30. Sheiner E, Levy A, Yerushalmi R, Katz M. Beta-thalassemia minor during pregnancy. Obstetrics & Gynecology. 2004;103(6):1273-7.

31. Shi H, Chen L, Wang Y, Sun M, Guo Y, Ma S, et al. Severity of Anemia During Pregnancy and Adverse Maternal and Fetal Outcomes. JAMA Network Open. 2022;5(2):e2147046-e.

32. Shumpert MN, Salihu H, Kirby R. Impact of maternal anaemia on birth outcomes of teen twin pregnancies: a comparative analysis with mature young mothers. Journal of Obstetrics and Gynaecology. 2004;24(1):16-21.

33. Smith C, Teng F, Branch E, Chu S, Joseph K. Maternal and perinatal morbidity and mortality associated with anemia in pregnancy. Obstetrics & Gynecology. 2019;134(6):1234-44.

34. Sun C-F, Liu H, Hao Y-H, Hu H-T, Zhou Z-Y, Zou K-X, et al. Association between gestational anemia in different trimesters and neonatal outcomes: a retrospective longitudinal cohort study. World Journal of Pediatrics. 2021;17:197-204.

35. Tzur T, Weintraub AY, Sergienko R, Sheiner E. Can anemia in the first trimester predict obstetrical complications later in pregnancy? J Matern Fetal Neonatal Med. 2012;25(11):2454-7.

36. Uta M, Neamtu R, Bernad E, Mocanu AG, Gluhovschi A, Popescu A, et al. The Influence of Nutritional Supplementation for Iron Deficiency Anemia on Pregnancies Associated with SARS-CoV-2 Infection. Nutrients. 2022;14(4).

37. Wilson NO, Ceesay FK, Hibbert JM, Driss A, Obed SA, Adjei AA, et al. Pregnancy outcomes among patients with sickle cell disease at Korle-Bu Teaching Hospital, Accra, Ghana: retrospective cohort study. The American journal of tropical medicine and hygiene. 2012;86(6):936.

38. Wu L, Sun R, Liu Y, Liu Z, Chen H, Shen S, et al. High hemoglobin level is a risk factor for maternal and fetal outcomes of pregnancy in Chinese women: A retrospective cohort study. BMC Pregnancy and Childbirth. 2022;22(1):290.
